# Frontotemporal Dementia and Suicide: A Scoping Review

**DOI:** 10.1093/geroni/igaf122.4127

**Published:** 2025-12-31

**Authors:** Samantha Tinker, Noelle Fields, Kathy Siepker, Jaclyn Kirsch, Soeun Jang

**Affiliations:** The University of Texas at Arlington, Arlington, Texas, United States; The University of Texas at Arlington, Arlington, Texas, United States; The University of Texas at Arlington, Arlington, Texas, United States; The University of Texas at Arlington, Arlington, Texas, United States; University of Texas at Arlington, The University of Texas at Arlington, Texas, United States

## Abstract

Persons living with frontotemporal dementia (FTD) have the highest suicide risk among persons living with dementia, yet their experiences are often overlooked in research and practice. This scoping review examined the literature on frontotemporal dementia and suicide, identified factors contributing to suicidal behavior, and explored potential treatment and prevention options. This encompassed the full spectrum of suicidality including suicidal ideation, attempts, and completed suicide. After formulating the research question, the researchers proceeded by following the PRISM-ScR guidelines. Five databases were systematically searched using Arksey and O’Malley’s framework. Studies were selected if they included FTD and suicide. Nineteen studies met the inclusion criteria. Key findings highlighted subtypes of FTD and suicide, risk factors in FTD and suicide, mental health conditions commonly occurring in FTD, and prevention and treatment methods. Instances of depression and other affective disorders were noted, alongside the characteristics and risk factors of suicide for the different subtypes of frontotemporal dementia. Findings underscore the need for prevention and treatment, family caregiver interventions, and the need for a tailored behavioral assessment for suicide in this population.

